# Case of Remarkable Presentation of the Fœtus in Utero

**Published:** 1871-11

**Authors:** Robert Anderson

**Affiliations:** M. D., L. R. C. S. Edin.


### Case of Remarkable Presentation of the Fœtus in Utero.

By Robert Anderson, M. D., L. R. C. S. Edin.

The following case came under my observation on the 25th of May, and presents many remarkable features, it being an instance in which, so far as I am aware, the greatest number of parts of the child presented at one and the same time. I therefore venture to think it may be of interest to the profession.

At 10 P. M., on the 25th of May I was summoned hurriedly to see Mrs. A——, who was in labour with her ninth child. She was being attended by a midwife, who, on finding something abnormal, sent at once for my assistance. She is a woman of very short statue, but with a roomy pelvis. She has had two still-born children previously at the full time, and also two footling presentations, both of which are alive. On my arrival I found she had been in labour since 8 A. M.; but with very light pains. The liquor amnii escaped about 4 P. M., and the pains had almost entirely ceased since then. On attempting to examine per vaginam, I discovered about nine inches of the cord outside the vulva, which I ascertained had existed for two or two hours. It was pulseless. When the finger was introduced into the regina, I found both feet presenting toward the posterior wall of the vagina; and, on sweeping round to feel the extent of dilatation of the os, I found a hand toward the right wall, and one toward the left. There was also a large, soft, bulging mass in the center, which, on first thought, I took for the breech; but, on pressing it, I found it to be some part of the head, with a large caput succedaneum. On finding the os fully dilated, I first endeavored to reduce the prolapsed cord; but failing, I seized both feet, and, bringing them down, delivery was easy. The child, of course, was dead; and all attempts at resuscitation were unavailing. The face of the child was delivered under the pubes, but proved no obstruction. An examination of the fœtus showed a caput succedaneum on the forehead, which was thus proved to have been the part of the head presenting, along with the cord, feet, and hands. The mother has progressed rapidly to recovery, and is now almost quite well.

The above case is to me singular as an instance of no fewer than six parts of the fœtus presenting in the vagina at once—viz., the umbilical cord, both feet, both hands, and the forehead. The face coming under the public arch was to be looked for when the position of the child in utero is considered. It however proving no hindrance to delivery, a very complicated presentation turned out a case of easy management.—*Lancet.*

